# Congenital heart defects of fetus after maternal exposure to organic and inorganic environmental factors: a cohort study

**DOI:** 10.18632/oncotarget.20110

**Published:** 2017-08-10

**Authors:** Wei Gong, Qianhong Liang, Dongming Zheng, Risheng Zhong, Yunjie Wen, Xiaodan Wang

**Affiliations:** ^1^ Department of Ultrasound, Panyu He Xian Memorial Hospital, Guangzhou City, Guangdong Province, 511400 China; ^2^ Guangzhou Huayin Medical Labratory Center. CO. Ltd, 510663 China

**Keywords:** congenital heart disease, pregnancy, organic solvent, noise

## Abstract

**Objective:**

Maternal exposure to various contaminants has been reported to be correlated with congetinal heart defects (CHDs). In this study, the effect of maternal exposure to organic and inorganic environmental factors upon the incidence of CHDs was investigated. We conducted a retrospective birth cohort study of infants born in the Maternal and Child Health Hospital of Panyu District in Guangzhou.

**Materials and Methods:**

A total of 5381 cases with complete medical records, including mothers, fathers, and infants, were enrolled. The relationship between maternal occupational exposure to hazardous substances and strong noise during pregnancy and CHDs was analyzed. Occupational exposure to hazardous substances increased the incidence of CHDs.

**Results:**

Forty-eight of 145 mothers (33.1%) in the CHDs group worked in hazardous and strong noise factories, while the corresponding percentage mothers in the control group was 22.8% (1193/5236). The percentage of mothers with a history of contact with organic solvents and exposure to strong noise in the CHDs group was significantly higher than the control group. There was no significant difference in the histories of contact with heavy metals, high temperatures, and other extreme environments between two groups.

**Conclusions:**

Hazardous substances in factories, especially organic solvents, were identified as potential risk factors for CHDs. Besides, exposure to high noise also increased the incidence of CHDs.

## INTRODUCTION

An industrialized lifestyle has led to a different human disease spectrum, which are technically preventable when the risk factors are identified. Maternal exposure to various contaminants has been reported to be associated with congenital heart defects (CHDs). Studies have shown that air pollution has a significant influence on CHDs, including intrauterine growth retardation, preterm births, and low birth weight [1–3]. Maternal exposure to air pollution is significantly associated with congenital anomalies [1, 4–13]. Maternal exposure to environmental pollution, such as arsenic and cadmium, also increase the risk of low birth weight and small birth size [14–16]. Thus, arsenic and cadmium have been identified as risk factors for congenital anomalies [17]. Smoke has also been shown to be a risk factor for congenital heart defects (CHDs) [18]. Atmospheric particulate matter, also known as particulate matter (PM), are microscopic solid or liquid matter suspended in Earth’s atmosphere. Subtypes of atmospheric particulate matter include suspended particulate matter (SPM), thoracic and respirable particles, inhalable coarse particles, which are particles with a diameter between 2.5 and 10 micrometers (μm) (PM10). High exposure to PM10 leads to musculoskeletal and chromosomal abnormalities, but has no effect on cardiovascular defects [19]. Indeed, these studies showed that maternal exposure to different contaminants during pregnancy is associated with different kinds of CHDs.

CHDs are the most common birth defect because the heart and vessels develop abnormally during embryogenesis or the channel does not close after birth. CHDs encompass a spectrum of pathologies, such as atrial septal defects (ASDs), ventricular septal defects (VSDs), patent ductus arteriosus (PDA), and tetralogy of Fallot (TOF). The cause of CHDs is also complicated, including genetic and environmental factors [20]. Approximately 20% of CHDs are due to known causes, such as genetic syndromes [21, 22]; the remaining 80% of CHDs have an unknown etiology. Studies have shown that the environment contributes significantly to the incidence of CHDs; however, the specific environmental factors that contribute to CHDs have not been determined. Identification of potential non-inherited risk factors for CHDs is essential in the elimination of precipitating factors and to decrease the incidence of CHD, which is of great public health significance. In this study, we investigated the exposure history to contaminants, such as organic and inorganic toxins before and during pregnancy, and analyzed the relationship between the exposure history and CHDs.

## MATERILAS AND METHODS

### Study population

In this observational cohort study, pregnant women admitted to Guangzhou Panyu Maternity Hospital were recruited. This study was approved by the Institutional Review Board of Guangzhou Panyu Maternity Hospital and written informed consents were obtained prior to data collection. A total of 5381 newborn records were reviewed with matched paternal information. A total of 5381 cases with complete medical records, including mother, father, and infant, were enrolled. The newborns were screened for CHDs and 145 CHD s cases were diagnosed.

### Exposure assessment

The exposure history of mothers to hazardous substances from 6 months before pregnancy to the prenatal examinations at 12, 20, 30, 36 and 40 weeks after pregnancy was investigated. Histories of working in chemical factories, and exposure to various organic solvents and heavy metals were investigated. The ionizing radiation and X-ray contact histories were also included.

### Statistical analysis

The data were evaluated for statistical differences using SAS9.4 software. All statistical tests were performed based on double-side inspection. A *P* value of less than 0.05 was considered as statistical significance.

## RESULTS

### General information of neonates

Of all 5381 newborns, 145 had CHDs, including malformations in the central nervous, digestive, and cardiovascular systems. The group with CHDs had an average gestational age of (35.01 ± 5.83) weeks, while the control group had an average gestational age of (38.60 ± 1.72) weeks; the difference was statistically significant (*t* = 7.375, *P* < 0.001, Table 1). The gender distribution significantly differed between two groups (*P* < 0.001). The medical records showed a significantly higher loss of gender identification, which was probably due to increased abortions chosen by parents before full term. The average weight of neonates with CHDs was (2525.47 ± 1050.56) g, while the control group had an average weight of (3152.08 ± 457.83) g; the difference was statistically significant (*t* = 7.039, *P* < 0.001; Table 1).

**Table 1:** Neonates general information

	Control (*n* = 5236)	CHDs (*n* = 145)	Total (*n* = 5381)	t/χ2	*P*
Sex, *n* (%)					
Female	2408 (46.1)	69 (47.6)	2477 (46.1)	76.063	< .001
Male	2804 (53.7)	69 (47.6)	2873 (53.5)		.
Not identified	14 (0.3)	7 (4.8)	21 (0.4)		.
Gestational age, week, Mean (SD)	38.60 (1.72)	35.01 (5.83)	38.51 (2.03)	7.375	< .001
Weight, gram, Mean (SD)	3152.08 (457.83)	2525.47 (1050.56)	3135.70 (492.71)	7.039	< .001
Number of birth, *n* (%)					
Single	5142 (98.6)	135 (93.1)	5277 (98.4)	38.329	< .001
Twins	74 (1.4)	9 (6.2)	83 (1.5)		.
Multiple	1 (0.0)	1 (0.7)	2 (0.0)		.
If more than single, *n* (%)					
Identical	13 (61.9)	3 (50.0)	16 (59.3)	0.274	0.601
Fraternal	8 (38.1)	3 (50.0)	11 (40.7)		.
Prognosis, *n* (%)					
Live	5193 (99.5)	112 (77.8)	5305 (98.9)	770.548	< .001
Die in uterus	8 (0.2)	1 (0.7)	9 (0.2)		.
Die after birth	1 (0.0)	0 (0.0)	1 (0.0)		.
Die within a week of birth	6 (0.1)	2 (1.4)	8 (0.1)		.
Abortion	11 (0.2)	29 (20.1)	40 (0.7)		.

### General information of mothers

The mothers of healthy neonates and the mothers with CHDs were then analyzed. The average age of mothers of healthy neonates was 27.1 ± 5.3 years, while the average age of mothers from the CHDs group was significantly higher (28.4 ± 5.3 years; *t* = −3.054, *P* = 0.002). The educational background of mothers also had a significant difference (χ^2^ = 11.448, *P* = 0.043); specifically, mothers of healthy neonates had a higher educational background. The family income of neonates with CHDs was also significantly lower than the healthy neonates (Table 2, 4138.66 ± 4223.54 vs, 3521.55 ± 2467.71, *t* = 2.896, *P* = 0.004). The other aspects did not show a significant difference between the control and CHDs groups (Table 2).

**Table 2:** General information of mothers

	Control (*n* = 5236)	CHDs (*n* = 145)	Total (*n* = 5381)	t/χ2	*P*
Age, year, Mean (SD)	27.1 (5.3)	28.4 (5.3)	27.1 (5.3)	−3.054	0.002
Ethnics, *n* (%)					
Han	5064 (97.7)	142 (98.6)	5206 (97.8)	0.485	0.486
Other	117 (2.3)	2 (1.4)	119 (2.2)		
Height, cm, Mean (SD)	158.50 (5.05)	158.14 (4.60)	158.49 (5.04)	0.832	0.406
Weight before pregnancy, kg, Mean (SD)	50.88 (8.29)	51.23 (8.12)	50.89 (8.29)	−0.500	0.617
Education, *n* (%)					
Primary	71 (1.4)	4 (2.8)	75 (1.4)	11.448	0.043
Mid-school	1419 (27.2)	52 (35.9)	1471 (27.4)		
Mid-associated degree	1640 (31.4)	42 (29.0)	1682 (31.4)		
associated degree	1281 (24.6)	33 (22.8)	1314 (24.5)		
Bachelor	741 (14.2)	11 (7.6)	752 (14.0)		
Graduate degree	65 (1.2)	3 (2.1)	68 (1.3)		
Monthly income, RMB, Mean (SD)	4138.66 (4223.54)	3521.55 (2467.71)	4122.03 (4186.93)	2.896	0.004

### Influence of exposure history to hazardous substances on CHDs

Comparing the occupational environment, 33.1% (48/145) of mothers in the CHDs group worked in harmful factories, while the percentage in the control group was 22.8% (1193/5236). The percentage was higher in the CHDs group than the control group (χ^2^ = 8.690, *P* = 0.013; Table 3). The percentage of mothers working in an extreme environment, non-extreme environment, and N/A in the CHDs group was 81.4% (118/145), 13.1% (19/145), and 5.5% (8/145), respectively. In the control group, the percentage of mothers working in an extreme environment, non-extreme environment, and N/A was 90.9% (4758/5236), 5.5% (288/5236), and 3.6% (187/5236) respectively. There was statistically different between the control and CHDs groups (χ^2^ = 17.163, *P* < .001). The percentage of mothers with a history of contact with organic solvents and exposure to strong noise in the CHDs group was higher than the control group. There was no significant difference in history of contact with heavy metals, high temperatures, and other extreme environments (Table 4).

**Table 3:** Maternal working history from 6 month before pregnancy to prenatal examination

	Control (*n* = 5236)	CHDs (*n* = 145)	Total (*n* = 5381)	t/χ2	*P*
Working history in following factories, *n* (%)					
No	3954 (75.7)	96 (66.2)	4050 (75.4)	8.69	0.013
Yes	1193 (22.8)	48 (33.1)	1241 (23.1)		.
N/A	76 (1.5)	1 (0.7)	77 (1.4)		.
Factories, *n* (%)					
Printing	71 (6.0)	2 (4.2)	73 (5.9)	0.265	0.606
Shoemaking	68 (5.7)	3 (6.3)	71 (5.7)	0.026	0.872
Electronics manufacturing	370 (31.0)	13 (27.1)	383 (30.9)	0.334	0.563
Chemical industry	27 (2.3)	2 (4.2)	29 (2.3)	0.733	0.392
Ferreteria	124 (10.4)	4 (8.3)	128 (10.3)	0.212	0.645
Furniture	39 (3.3)	1 (2.1)	40 (3.2)	0.208	0.648
Plastic product industry	60 (5.0)	1 (2.1)	61 (4.9)	0.857	0.355
Toy factory	43 (3.6)	2 (4.2)	45 (3.6)	0.042	0.838
Insecticide Factory	1 (0.1)	0 (0.0)	1 (0.1)	0.04	0.841
Pulp Mill	6 (0.5)	0 (0.0)	6 (0.5)	0.243	0.622
Dyeing Mill	11 (0.9)	1 (2.1)	12 (1.0)	0.65	0.42
Oil Refinery	2 (0.2)	0 (0.0)	2 (0.2)	0.081	0.776
Tannery	19 (1.6)	1 (2.1)	20 (1.6)	0.07	0.791
Ceramics Factory	4 (0.3)	0 (0.0)	4 (0.3)	0.161	0.688
Other factories	368 (30.8)	21 (43.8)	389 (31.3)	28.817	< .001

**Table 4:** Maternal working history in extreme environment and exposure history to hazardous materials from 6 month before pregnancy to prenatal examination

	Control (*n* = 5236)	CHDs (*n* = 145)	Total (*n* = 5381)	t/χ2	*P*
Working in extreme environment, *n* (%)					
No	4758 (90.9)	118 (81.4)	4876 (90.7)	17.163	< .001
Yes	288 (5.5)	19 (13.1)	307 (5.7)		.
N/A	187 (3.6)	8 (5.5)	195 (3.6)		.
Working environment					
High temperature, *n* (%)	17 (5.9)	1 (5.3)	18 (5.8)	0.012	0.911
Strong noise, *n* (%)	53 (18.3)	8 (42.1)	61 (19.8)	6.34	0.012
High dust, *n* (%)	28 (9.7)	0 (0.0)	28 (9.1)	2.025	0.155
Smell, *n* (%)	131 (45.3)	6 (31.6)	137 (44.5)	1.365	0.243
Exposure to chemicals, *n* (%)	87 (30.1)	4 (21.1)	91 (29.5)	0.702	0.402
Heavy metal, *n* (%)	14 (4.8)	0 (0.0)	14 (4.5)	0.964	0.326
Ionization radiation, n (%)	12 (4.2)	1 (5.3)	13 (4.2)	0.054	0.816
Contact to organic solvent, *n* (%)					
No	4800 (91.7)	123 (84.8)	4923 (91.5)	8.91	0.012
Yes	239 (4.6)	11 (7.6)	250 (4.6)		.
N/A	196 (3.7)	11 (7.6)	207 (3.8)		.
Organic solvent					
Diluent, *n* (%)	12 (5.0)	1 (9.1)	13 (5.2)	0.358	0.549
Thinner, *n* (%)	82 (34.2)	3 (27.3)	85 (33.9)	0.223	0.637
Gelatin,n (%)	54 (22.5)	1 (9.1)	55 (21.9)	1.105	0.293
White gas, n (%)	20 (8.3)	1 (9.1)	21 (8.4)	0.008	0.929
Cleaning agent, n (%)	36 (15.0)	1 (9.1)	37 (14.7)	0.292	0.589
Scaling powder, *n* (%)	10 (4.2)	0 (0.0)	10 (4.0)	0.477	0.49
Passivator, *n* (%)	3 (1.3)	0 (0.0)	3 (1.2)	0.139	0.709
Rust preventive oil, *n* (%)	11 (4.6)	0 (0.0)	11 (4.4)	0.527	0.468
Oil paint, n (%)	34 (14.2)	0 (0.0)	34 (13.5)	1.802	0.179
Alcohol, n (%)	85 (35.4)	6 (54.5)	91 (36.3)	1.665	0.197
Engine oil, *n* (%)	16 (6.7)	0 (0.0)	16 (6.4)	0.783	0.376
Ink, *n* (%)	18 (7.5)	1 (9.1)	19 (7.6)	0.038	0.845
Fuel, *n* (%)	4 (1.7)	1 (9.1)	5 (2.0)	2.969	0.085
Others, *n* (%)	2 (0.8)	0 (0.0)	2 (0.8)	0.092	0.761

The investigation of other risk factors showed that living in a newly decorated house, the distance between house-to-road, and pollution sources other than the house had no significant influence on CHDs. There was also no difference between the control and CHDs groups with respect to histories of exposure to X-rays, electromagnetic radiation, and ionization radiation (Table 5).

**Table 5:** Maternal contact history to other risk factors from 6 month before pregnancy to prenatal examination

	Control (*n* = 5236)	CHDs (*n* = 145)	Total (*n* = 5381)	t/χ2	*P*
Living in new decorating house, *n* (%)					
No	4704 (89.9)	127 (87.6)	4831 (89.8)	0.794	0.373
Yes	531 (10.1)	18 (12.4)	549 (10.2)		.
Distance from house to road, *n* (%)					
< 20 meter	428 (8.2)	7 (4.8)	435 (8.1)	2.326	0.313
20–50 meter	871 (16.7)	27 (18.6)	898 (16.7)		.
> 50 meter	3932 (75.2)	111 (76.6)	4043 (75.2)		.
Pollution sources besides the house, *n* (%)					
No	4949 (94.6)	134 (92.4)	5083 (94.6)	1.345	0.246
Yes	281 (5.4)	11 (7.6)	292 (5.4)		.
X-ray, *n* (%)					
No	5033 (96.2)	141 (97.2)	5174 (96.2)	0.938	0.626
Yes	30 (0.6)	0 (0.0)	30 (0.6)		.
N/A	168 (3.2)	4 (2.8)	172 (3.2)		.
Electromagnetic radiation, *n* (%)					
No	4774 (91.2)	130 (89.7)	4904 (91.2)	1.565	0.457
Yes	130 (2.5)	6 (4.1)	136 (2.5)		.
N/A	328 (6.3)	9 (6.2)	337 (6.3)		.
Ionization radiation, *n* (%)					
No	4972 (95.0)	138 (95.2)	5110 (95.1)	0.085	0.959
Yes	3 (0.1)	0 (0.0)	3 (0.1)		.
N/A	256 (4.9)	7 (4.8)	263 (4.9)		.

## DISCUSSION

Epidemiologic studies have shown that maternal exposure to harmful environmental factors had a significant association with birth defects in offspring, including low weight, growth retardation, and CHDs. These environmental factors include physical, chemical, and biological factors. We conducted a retrospective birth cohort study involving infants and found that the maternal occupational environment and contact history to different hazardous substances from 6 months before pregnancy to the prenatal examination had a different influence on the incidence of CHDs.

With industrial progress, environmental pollution has become more and more serious. Harmful substances are released from different factories. Industrialized lifestyle has led to a different human disease spectrum. Occupational exposure to toxic substances, such as pesticides, dyes, and oil paints were significantly associated with CHDs. We investigated the working history of mothers from 6 months before pregnancy to the prenatal examination and found that the percentage of CHDs was higher in factories with harmful substances. Forty-eight of 145 mothers (33.1%) in the birth defect group worked in factories with harmful substances, while the percentage in the control group was 22.8% (1193/5236). Different factories release different substances; however, there was no significant difference when we compared the factories, such as printing, electronic manufacturing, and furniture.

To further investigate the possible risk factors in these factories, organic solvents and heavy metals were selected as targets. Organic solvents are a chemical class of compounds that are widely used in factories, such as paint manufacturing, spray painting, degreasing, and metal processing. Studies of maternal occupational exposure to organic solvents showed an increased risk of ventricular septal and conotruncal defects [23, 24]. Thus, we speculated that organic solvents in these factories were correlated with CHDs. We indeed found that the percentage of mothers with a history of contact to organic solvents in the birth defect group was higher than the control group. Heavy metals are also pollution substances in these factories. Studies involving heavy metal exposure on CHD showed that maternal exposure to arsenic and cadmium was a significant risk factor for CHDs [17]. Maternal lead exposure is associated with the risk of some subtypes of CHDs [25]; however, there was no significant influence of heavy metals on CHDs. Therefore, we inferred that the potential risk factors in these harmful factories were organic solvents. With respect to heavy metals, the incidence of CHDs may be related to the low contact rate of the subjects. The influence of heavy metals on CHDs warrants further investigation.

In addition to harmful factories, we also investigated working in extreme environments, such as high temperatures, high noise, high dust, odors, and radiation. We found that high noise was related to CHDs; however, high temperatures and radiation had no influence on the CHD rate. The studies involving high temperature on CHDs had different conclusions. Animal studies showed that hyperthermia was related to malformations [26]. Agay-Sha et al. [27] assessed the influence of maternal exposure to average ambient temperature and extreme heat events on CHDs and reported that there was no significant relationship between ambient temperature and CHDs; however the extreme heat events increased the risk for CHDs. Tikkanen [28] reported that temperature at the workplace was not a risk factor for CHDs. Maternal exposure to microwave ovens, disinfectants, pesticides, or anesthetic gases were not associated with CHDs.

We also investigated the general exposure to physical and chemical factors, such as a newly decorated house, the distance between house to road and pollution sources besides the house. X-ray, electromagnetic radiation and ionization radiation were also investigated. There was no different between control group and CHDs group.

In conclusion, we investigated the influence of maternal occupational exposure to organic and inorganic environmental factors during pregnancy on CHDs. Hazardous substances in factories, especially organic solvents, were identified as potential risk factors for CHDs. High noise also increased the incidence of CHDs.
